# Pilot Study of Essential Drug Quality in Two Major Cities in India

**DOI:** 10.1371/journal.pone.0006003

**Published:** 2009-06-23

**Authors:** Roger Bate, Richard Tren, Lorraine Mooney, Kimberly Hess, Barun Mitra, Bibek Debroy, Amir Attaran

**Affiliations:** 1 Africa Fighting Malaria, Washington, D. C., United States of America; 2 American Enterprise Institute, Washington, D. C., United States of America; 3 Africa Fighting Malaria, Cambridge, United Kingdom; 4 Asia Fighting Malaria, Delhi, India; 5 Centre for Policy Research, Delhi, India; 6 Institute of Population Health and Faculties of Law and Medicine, University of Ottawa, Ottawa, Canada; McGill University Health Center, Montreal Chest Institute, Canada

## Abstract

**Background:**

India is an increasingly influential player in the global pharmaceutical market. Key parts of the drug regulatory system are controlled by the states, each of which applies its own standards for enforcement, not always consistent with others. A pilot study was conducted in two major cities in India, Delhi and Chennai, to explore the question/hypothesis/extent of substandard and counterfeit drugs available in the market and to discuss how the Indian state and federal governments could improve drug regulation and more importantly regulatory enforcement to combat these drugs.

**Methodology/Principal Findings:**

Random samples of antimalarial, antibiotic, and antimycobacterial drugs were collected from pharmacies in urban and peri-urban areas of Delhi and Chennai, India. Semi-quantitative thin-layer chromatography and disintegration testing were used to measure the concentration of active ingredients against internationally acceptable standards. 12% of all samples tested from Delhi failed either one or both tests, and were substandard. 5% of all samples tested from Chennai failed either one or both tests, and were substandard. Spatial heterogeneity between pharmacies was observed, with some having more or less substandard drugs (30% and 0% respectively), as was product heterogeneity, with some drugs being more or less frequently substandard (12% and 7% respectively).

**Conclusions/Significance:**

In a study using basic field-deployable techniques of lesser sensitivity rather than the most advanced laboratory-based techniques, the prevalence of substandard drugs in Delhi and Chennai is confirmed to be roughly in accordance with the Indian government's current estimates. However, important spatial and product heterogeneity exists, which suggests that India's substandard drug problem is not ubiquitous, but driven by a subset of manufacturers and pharmacies which thrive in an inadequately regulated environment. It is likely that the drug regulatory system in India needs to be improved for domestic consumption, and because India is an increasingly important exporter of drugs for both developed and developing countries. Some poor countries with high burdens of disease have weak drug regulatory systems and import many HIV/AIDS, tuberculosis and malaria drugs from India.

## Introduction

India presents definite opportunities and potential perils to global health in its prolific pharmaceutical industry, for India is a leading supplier of high quality generic drugs throughout the world, but it is also a leading source of counterfeit drugs [1].

Substandard and counterfeit drugs have grave consequences for public health. Drugs with too little or no active ingredient can cause patient death and lead to the development of drug resistance. Resistance at the population level renders legitimate drugs and even entire classes of drugs less effective, even for patients who did not previously take poor-quality drugs.

India has a self-admitted problem of manufacturing unreliable drugs. In 2002, the World Health Organisation (WHO) reported that Indian pharmaceutical manufacturers themselves estimated 20% of drugs in major Indian-city markets were substandard or illegal [2]. Similarly, the Indian government estimates that counterfeit drugs account for 0.34% of the total pharmaceutical market and substandard drugs account for 9.34% [3]. These data are based on samples tested by the state authorities between 1995 and 2003; the extent of substandard drugs varied from 8.19 to 10.64 percent and counterfeit drugs varied between 0.24 and 0.47 percent [3]. Other evidence suggests that the quality of India's drugs has a global impact. In May 2008, some of the authors published a study assessing the quality of antimalarial drugs in Africa [4]. The study found that 35% of antimalarial drugs sold in private shops and pharmacies in six major African cities failed basic quality control tests [4]. 31% of the samples purportedly of Indian origin were found to be substandard.

In 2003, in response to the growing menace of counterfeit drugs, the Indian government commissioned an expert committee under Dr. R.A. Mashelkar to examine the counterfeit drug problem and to devise a reform of the regulatory system in India. The Committee's report identified many failings, but found that diligence varied markedly between India's states. For example, 17 of 31 states and united territories that responded had functional drug testing laboratories, of which only seven were adequately equipped and staffed [3]. The Committee recommended upgrading India's regulations and regulatory bodies to meet international standards, to include a National Drug Authority.

The WHO recommends that each country have a “central coordinating body with overall responsibility and accountability for all aspects of drug regulation for the entire country [5].” The creation of a National Drug Authority has been proposed several times in India but has yet to be enacted.

In 2004, drug-industry analysts estimated that with a compound annual growth rate between 7 and 10 percent, India's pharmaceutical industry would be worth between $6 and $7 billion in 2008. Given the increasing importance of India as a global supplier of drugs and active pharmaceutical ingredients for treatment of all diseases, it is important to improve certainty about the quality of drugs manufactured in India.

Randomly selected samples were collected from randomly chosen pharmacies scattered in and around Delhi and Chennai. Samples of the following drugs listed on the WHO Model List of Essential Medicines were procured: chloroquine, ciprofloxacin, erythromycin, isoniazid and rifampicin. This paper assesses the quality of a small selection of these primarily domestically-produced drugs on sale in pharmacies in Delhi and Chennai in an attempt to determine the prevalence of substandard drugs available to city residents. A total of 541 random samples procured from 52 pharmacies were assessed using basic field screening techniques for drug quality.

## Results

The drugs procured in this study were readily available over-the-counter without a prescription in Delhi and Chennai pharmacies, including all anti-infectives shown in Table 1.

**Table 1:** Testing results by drug type and location for TLC and disintegration.

		Number failing TLC	Number failing disintegration	Number failing TLC or disintegration	Number tested	Percent failing TLC or disintegration
Ciprofloxacin	Delhi	5	5	5	50	10%
	Chennai	3	1	3	53	6%
	Combined Total	8	6	8	103	8%
Chloroquine	Delhi	5	5	5	56	9%
	Chennai	3	1	3	63	5%
	Combined Total	8	6	8	119	7%
Erythromycin	Delhi	8	8	8	61	13%
	Chennai	1	1	1	56	2%
	Combined Total	9	9	9	117	8%
Isoniazid	Delhi	6	8	8	48	17%
	Chennai	1	1	2	36	6%
	Combined Total	7	9	10	84	12%
Rifampicin	Delhi	8	7	8	66	12%
	Chennai	3	2	3	52	6%
	Combined Total	11	9	11	118	9%
**TOTAL**	**Delhi**	**32**	**33**	**34**	**281**	**12%**
	**Chennai**	**11**	**6**	**12**	**260**	**5%**
	**Combined**	**43**	**39**	**46**	**541**	**8.5%**

Samples from 281 treatment packs collected from Delhi pharmacies were tested in duplicate in July 2008, comprising 50 ciprofloxacin, 56 chloroquine, 61 erythromycin, 48 isoniazid and 66 rifampicin. Having recorded solely the better-performing sample in the duplicate pair, which is a generous assumption that may understate the incidence of poor drug quality, 12% (34/281) of tested samples failed thin-layer chromatography (TLC) and/or disintegration tests. The breakdown of failures is as follows: 0.7% (2/281) failed only disintegration tests, 0.4% (1/281) failed only TLC, and 11% (31/281) failed both TLC and disintegration tests (See Table 1). 10% of ciprofloxacin, 9% of chloroquine, 13% of erythromycin, 17% of isoniazid and 12% of rifampicin failed one or more tests.

Samples from 260 treatment packs collected from Chennai pharmacies were tested in duplicate in March 2009, comprising 53 ciprofloxacin, 63 chloroquine, 56 erythromycin, 36 isoniazid and 52 rifampicin. Having again recorded the better-performing sample in the duplicate pair, 5% (12/260) of tested samples failed TLC and/or disintegration tests. The breakdown of failures is as follows: 0.4% (1/260) failed only disintegration tests, 2% (6/260) failed only TLC, and 2% (5/260) failed both TLC and disintegration tests (See Table 1). 6% of ciprofloxacin, 5% of chloroquine, 2% of erythromycin, 6% of isoniazid and 6% of rifampicin failed one or more tests.

In total, 541 samples were collected from pharmacies in Delhi and Chennai, with 8.5% (46/541) of tested samples failing TLC and/or disintegration tests.

The authors did not conduct forensic analysis of the drugs to determine whether they were substandard or counterfeit, as results of previous attempts to collect valid samples and batch information from companies for comparative examination were only partly successful. However, fewer than 4% (11/281) of samples collected in Delhi had zero active ingredients and only two samples collected in Chennai had very low concentrations of active ingredients, both of which are indicators of counterfeit provenance. Assuming the country of origin stated on the drug packaging was correct, 97% (524/541) of tested samples were manufactured in India, of which 8% (42/524) failed the above quality control tests. Of these, 21% (9/42) had zero or very low concentrations of active ingredients. 3% (17/541) of tested samples (all from Delhi) were labeled as manufactured in the United States, of which 23.5% (4/17) failed basic quality control tests. All four U.S. samples that failed one or more tests had zero active ingredients, suggesting they could be counterfeit.

**Figure 1:**
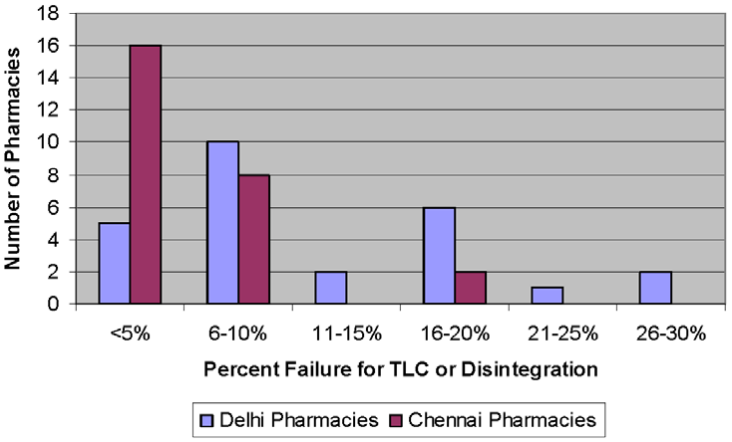
Percentage of tested samples failing TLC or disintegration by number of pharmacies sampled in Delhi and Chennai (all pharmacies with fewer than 5% failures actually had zero failures).

Of the 26 pharmacies sampled in Delhi, five pharmacies had no failures, while seven had from 20 to 30 percent failures (See Figure 1); these seven pharmacies also supplied 10 of the 11 samples found to contain zero active ingredients. Of the 26 pharmacies sampled in Chennai, 16 pharmacies had no failures and none of the pharmacies sampled had failures above 20% (See Figure 1).

Given the small sample size in this study, similar studies of the same drugs from other cities in other states would allow an assessment of whether those states with better regulatory environments and/or testing regimes have better quality drugs. Further analysis of the behavior of pharmacists, notably their drug procurement practices, is also warranted, given the variation in drug quality found from different pharmacies around Delhi and Chennai.

## Discussion

According to the World Malaria Report 2008, there were an estimated 10.6 million cases of malaria in India in 2006 [6]. Chloroquine is still the main treatment for uncomplicated malaria in India. The WHO ranks India first in terms of total numbers of cases of tuberculosis (TB). In 2007, there were more than 1.9 million new cases of TB in India [7]. Isoniazid and rifampicin are considered powerful first line drugs for treatment of TB.

At a 12% failure rate, the quality of drugs in peri-urban pharmacies around Delhi is slightly worse than the Indian government's estimate (roughly 10%) and better than that estimated by the Indian pharmaceutical industry, as reported to the WHO. The quality of drugs in pharmacies around Chennai is slightly better, with only a 5% failure rate. Although the sample sizes are too small to make broad conclusions and/or generalizations about the quality of essential drugs in India, they provide at least one reasonable scenario of the cities sampled. The failures could be the result of deliberate counterfeiting, or substandard production, transport or storage. The authors discussed these results with several local counterfeit drug investigators. The conclusions of these discussions were as follows: The wide variation in failure rates among pharmacies (See Figure 1) suggests that most pharmacists are buying good quality drugs and storing them properly. However, some pharmacists are either buying, wittingly or unwittingly, substandard drugs, expired drugs that have had their packaging possibly re-stamped with new expiry dates, or are incapable or unwilling to store drugs correctly.

The 12% and 5% failure rates in Delhi and Chennai respectively are perhaps lower than expected, given that some WHO-reported data indicates it to be higher [2]. This variation calls for a system of enforcement to maintain a certain minimum quality criteria. Given the heavy burden of TB and malaria in the country, it is pertinent that high quality drugs be made available to residents. A similar system for exporting drugs should also be in place.

The failure rates observed in this study could be good news, demonstrating improved performance driven by the Indian authorities in response to increased awareness of the regulatory gaps as revealed by the Mashelkar Report in 2003. However, it should be noted that the Indian government has called upon committees in the past to review the regulatory system and make recommendations for its improvement but “these recommendations have been implemented by the Government to some extent, but the core issues have remained unresolved [3].” The improvement in failure rates could also be the result of individuals, such as the recent Indian Health Minister, Anbumani Ramadoss from Tamil Nadu state (capital Chennai), who has championed combating substandard drugs and may have also contributed to the better performance of Chennai-sourced drugs in this study.

Sampled pharmacies were located in urban and peri-urban areas of Delhi and Chennai, which have both higher wealth (double the national average in Delhi) and more tightly enforced regulation compared with more rural areas. Regardless, a 12% or 5% failure rate of essential drugs poses a significant threat to the respective health of Delhi and Chennai residents, and if the results are indicative of the country as a whole, there is an even greater danger to far more people. Assuming all cases of malaria and TB were treated, even a 1% failure rate would mean over 100,000 patients would receive substandard drugs. If the failure rates observed in this study were indicative of the country as a whole, then just over a million patients would access substandard drugs for just these two diseases. This extrapolation is a likely scenario if this problem is not addressed.

Why are substandard drugs prevalent in India? The Mashelkar Report of 2003 noted, “the problems in the regulatory system in the country were primarily due to inadequate or weak drug control infrastructure at the State and Central level, inadequate testing facilities, shortage of drug inspectors, non-uniformity of enforcement, lack of specially trained cadres for specific regulatory areas, non-existence of data bank and nonavailability of accurate information [3].” In addition, the division of labor between the central and state regulatory agencies creates inconsistencies in regulatory requirements and policies across the country. Individual states are responsible for licensing and monitoring domestic drug manufacturers for quality, and pursuing legal action against offenders. This federal structure means that India lacks national norms for drug quality, and that most of the quality policing is done at state level without uniformity of action. This means a manufacturer producing substandard drugs could receive approval in a state with weak controls, and its drugs could be sold anywhere in the country.

Other shortcomings of India's regulatory system include poor enforcement and outdated legislation. Legal proceedings are “far too complicated and lengthy; the process moves slowly and the conviction rate is low [3].” The 1940 Drugs and Cosmetics Act, which governs drug production and sale in India, “contains various provisions for effective punitive action against manufacturers and distributors [3].” For adulterated drugs that likely caused death or bodily harm, the Act's lack of specificity has led to its under-utilization.

Additionally, there are too few drug inspectors in India, while the number of licensed manufacturing and selling premises increases exponentially. Pharmacists are not required to register with professional bodies or state boards and do not need to continue education after their initial qualification. Furthermore, there is a strong culture of self-prescription in the country, enabled by pharmacists willing to sell drugs without a prescription.

There are some important ramifications for foreign buyers of Indian drugs. If within India's own borders, and in relatively prosperous cities (Delhi and Chennai), a proportion (12% and 5% respectively) of drugs are substandard for various reasons, an assumption of safety is probably premature for at least some of the drugs that India exports. While developed nations provide strict import quality controls, probably screening out suspect products, this is not the case for most of the developing world. At present, Indian state drug regulatory authorities issue export licenses as well as a Certificate of Pharmaceutical Product to facilitate such trade. In all probability this is insufficient government oversight. Dora Akunyili, former Director General of Nigeria's National Agency for Food and Drug Administration and Control, routinely complained about drugs coming into Nigeria from India and banned at least 25 Indian drug companies from exporting to Nigeria [8].

The Indian government should lead an effort to harmonize regulatory requirements across states and consolidate regulatory functions, as recommended by the WHO. It should also increase penalties for those involved in the sale of substandard products as recommended by the Mashelkar Committee. It could also consider requiring that all drug manufacturers receive licenses from the central government, even while allowing states with sufficient regulatory capacity to continue licensing sales establishments.

This pilot study calls for a larger study to explore the extent of the availability of substandard and counterfeit drugs in the market. It calls for an investigation of pharmacies located in different parts of the country - rural, semi-urban, peri-urban - where the problem may be more rampant due to less stringent drug quality enforcement. It also calls for an internal Indian review of the counterfeit drug problem to tackle the TB, HIV/AIDS and malaria epidemics in the country.

## Materials and Methods

The simple sampling protocol was developed in line with previously published research [4], [9]. Indian nationals from Delhi and Chennai posed as customers and made drug purchases from storefront pharmacies located in middle-class areas of each city. The “customers” were instructed to stay within a single neighborhood and to select pharmacies at first sight on a random walk, and were blind as to the purpose for which they were collecting samples. Samples were obtained in June 2008 from 26 randomly selected private pharmacies in urban and peri-urban areas of Delhi (the capital located in northern India). Samples were also obtained in March 2009 from 26 randomly selected private pharmacies in urban and peri-urban areas of Chennai (the largest city in southeast India). “Customers” purchased a sample lot of an antimalarial (chloroquine, which remains the norm for treating *Plasmodium vivax* malaria in Delhi and Chennai), two antibiotics (ciprofloxacin and erythromycin) and two antimycobacterials (isoniazid and rifampicin) without a prescription. All of these drugs are included on the WHO Model List of Essential Medicines. Treatment packs included drugs sold in the manufacturer's original packaging as well as those distributed loose, often in paper bags. 2% of collected samples were sold loose with roughly the same quality performance (one failure out of ten samples tested) as those in blister packs. Once purchased, all drugs were stored at ambient temperature, with low humidity and no sunlight – until testing. Tests were completed within 40 days of sample collection.

The Global Pharma Health Fund e.V. Minilab was used to run semi-quantitative thin-layer chromatography and disintegration tests on each sample to determine the presence and relative concentration of active ingredients [10]. The Minilab® protocols award products a “pass” for TLC if 80% or more of the labeled active ingredient(s) is present. Samples were also tested to see if they disintegrated in water at 37°C in less than 30 minutes. Each test was run in duplicate, with the generous assumption that the result more consistent with the reference was recorded. Quality control of the Minilab was performed daily prior to drug testing and consisted of performing TLC on Minilab-reference samples for the five drug classes being analyzed. In addition, Minilab reagents were quality control tested using reference samples when a new lot was introduced.
